# Gender difference in mortality among pulmonary tuberculosis HIV co-infected adults aged 15-49 years in Kenya

**DOI:** 10.1371/journal.pone.0243977

**Published:** 2020-12-14

**Authors:** Rose J. Kosgei, Steven Callens, Peter Gichangi, Marleen Temmerman, Anne-Beatrice Kihara, Gathara David, Eunice Nyaboe Omesa, Enos Masini, E. Jane Carter

**Affiliations:** 1 Department of Obstetrics and Gynaecology, University of Nairobi, Nairobi, Kenya; 2 Ghent University, Faculty of Medicine and Health Sciences, Ghent, Belgium; 3 Department of Human Anatomy, University of Nairobi, Nairobi, Kenya; 4 Aga Khan University, Faculty of Health Sciences, Nairobi, Kenya; 5 Ruby Medical Centre, Kiambu West, Kenya; 6 National Tuberculosis Leprosy and Lung Disease Program, Nairobi, Kenya; 7 Alpert School of Medicine at Brown University, Providence, Rhode Island, United States of America; National Institute for Communicable Disease (NICD), South Africa, SOUTH AFRICA

## Abstract

**Setting:**

Kenya, 2012–2015

**Objective:**

To explore whether there is a gender difference in all-cause mortality among smear positive pulmonary tuberculosis (PTB)/ HIV co-infected patients treated for tuberculosis (TB) between 2012 and 2015 in Kenya.

**Design:**

Retrospective cohort of 9,026 smear-positive patients aged 15–49 years. All-cause mortality during TB treatment was the outcome of interest. Time to start of antiretroviral therapy (ART) initiation was considered as a proxy for CD4 cell count. Those who took long to start of ART were assumed to have high CD4 cell count.

**Results:**

Of the 9,026 observations analysed, 4,567(51%) and 4,459(49%) were women and men, respectively. Overall, out of the 9,026 patients, 8,154 (90%) had their treatment outcome as cured, the mean age in years (SD) was 33.3(7.5) and the mean body mass index (SD) was 18.2(3.4). Men were older (30% men’ vs 17% women in those ≥40 years, p = <0.001) and had a lower BMI <18.5 (55.3% men vs 50.6% women, p = <0.001). Men tested later for HIV: 29% (1,317/4,567) of women HIV tested more than 3 months prior to TB treatment, as compared to 20% (912/4,459) men (p<0.001). Mortality was higher in men 11% (471/4,459) compared to women 9% (401/4,567, p = 0.004). There was a 17% reduction in the risk of death among women (adjusted HR 0.83; 95% CI 0.72–0.96; p = 0.013). Survival varied by age-groups, with women having significantly better survival than men, in the age-groups 40 years and over (log-rank p = 0.006).

**Conclusion:**

Women with sputum positive PTB/HIV co-infection have a significantly lower risk of all-cause mortality during TB treatment compared to men. Men were older, had lower BMI and tested later for HIV than women.

## Introduction

Gender disaggregation of data in tuberculosis (TB) and human immunodeficiency virus (HIV) co-infection is important, for patients in reproductive age (15–49 years) for equity and equality in program service delivery [1]. In TB/HIV co-infected patients, survival depends on timely initiation of both anti-TB and antiretroviral therapy (ART) treatment [2]. In 2016, there were about 6.3 million new cases of TB reported globally, out of which 476,774 (8%) were co-infected with HIV [3]. Tuberculosis is the leading cause of death in patients living with HIV in resource limited settings [4, 5]. A sub-analysis of a recent study in Kenya that included patients with pulmonary TB (PTB) regardless of their HIV status [6] concluded that women of reproductive age had a worse outcome of PTB treatment compared to age-matched men, attributed to HIV infected patients who were not on ART while on TB treatment.

Studies have demonstrated a clear mortality reduction and improved outcome when ART was initiated, regardless of CD4 cell count in all TB/HIV infected patients during TB treatment [2]. However, due to the risk of immune reconstitution inflammatory syndrome (IRIS) when ART is initiated in the early phase of TB treatment, current recommendations suggest ART initiation between 14 days and 60 days after the anti-TB treatment initiation [7–9]. Previous work has shown the incidence of TB IRIS in TB/HIV co-infected patients to be 8–43% of TB cases [4, 10]. The risk factors for IRIS are low CD4 cell counts, reduced CD4/CD8 ratio, low haemoglobin level, low body mass index (BMI) and disseminated TB disease [4].

The Kenya’s National Tuberculosis and Lung Diseases Program (NTLD-P), and the National AIDS Control Program (NASCOP), recommend initiation of ART 14 days after anti-TB treatment. Despite this recommendation, there is anecdotal evidence that not all TB/HIV co-infected patients are promptly initiated on ART during TB treatment. Studies from Africa, Cambodia and Vietnam have identified barriers to initiation of ART among TB/HIV infected patients to include; lack of integration of HIV and TB services, young age, being a man, overlapping drug toxicities and local policies of TB/HIV programs [5, 11, 12].

The current study sought to explore whether there is a gender difference in all-cause mortality among smear positive PTB/HIV co-infected patients treated for TB between 2012 and 2015 in Kenya.

## Methods

### Setting

The NTLD-P has a national case-based electronic data capturing system referred to as “Tuberculosis Information from Basic Units“(TIBU which also means treatment in Swahili). The system is based on the World Health Organization standard TB definitions and reporting framework [13]. Individual patient data is captured in the TIBU system at the health facility and relayed to a national database [6]. All public, faith-based, and private treatment centres in the country enter individual-level data into the centrally located system. TIBU was rated ‘highly valuable’ for monitoring, evaluation, support supervision and data quality in 2012 [14].

The TB data from all the 47 counties in Kenya were analysed. An earlier study in Kenya showed varying PTB treatment outcomes across the 47 counties [6]. This was attributed mainly to differences in demographics, epidemiology, HIV prevalence and socioeconomic status [15]. Integration of TB and HIV services are not uniform across all facilities. Some TB treatment facilities (mostly hospitals) are fully integrated and offer HIV testing and ART to TB/HIV co-infected patients. Few, lower level facilities are partially integrated, mainly offering only HIV testing but not treatment and care. Treatment of TB and registration into TIBU is based on either bacteriological confirmation (smear, GenXpert or culture positive) or clinical diagnosis (suggestive radiology, suggestive histopathology and extrapulmonary TB). At the time of the study, the NTLD-P recommendation was to initiate ART in TB-HIV co-infected patients, if CD4 cell counts were less than 500 cells/μl, and the first line anti-TB regimen was Isoniazid (H), Rifampicin (R), Pyrazinamide (Z), Ethambutol (E) and Streptomycin (S); given as two months intensive phase of RHZE and four months continuation phase of RH (2RHZE/4RH).

### Study design

The study was a retrospective cohort analysis of NTLD-P data.

### Study population

The study population was composed of smear positive PTB/HIV co-infected patients aged between 15–49 years treated for TB between 2012 and 2015 (n = 29,592). This database did not include multi-drug resistant TB patients which are captured in a different database. Patients who transferred out and those still on treatment at the time of analysis were excluded. Patients who were coded ‘as out of control’ were renamed to ‘lost to follow-up’ consistent with the revised WHO definitions and reporting framework for TB [16]. The primary outcome of the study was patients who were either bacteriologically confirmed as cured or those who died. As such, patients whose outcome was documented as ‘treatment completed but no bacteriological confirmation of outcome data’, those who ‘failed treatment’ and those who ‘were lost to follow-up’ were excluded. Additionally, since the study interest was to explore the effect of the temporal sequence on the outcome for duration and timing of HIV test to start of TB treatment and timing of ART initiation, all patients who had; missing data on HIV testing date, TB treatment start date and ART start date were excluded. However, the excluded patients were considered in the sensitivity analysis.

### Variables

The outcome variable was all-cause mortality during TB treatment defined as Death (Yes/No). However, for those “Death = No” we only considered those documented as “Cured”; defined as a PTB patient with bacteriologically confirmed TB at the beginning of treatment who was smear or culture negative, both in the last month of treatment or on at least one previous occasion, where the last month results were not available. “Died” was defined as a TB patient who died for any reason before starting (but was registered on TIBU) or during treatment. The primary exposure variable was gender (men/women).

The following exposure variables were extracted from the TIBU dataset: age, BMI, year of treatment enrolment to the TB program, gender, sputum smear at month 2, 5 and 6, anti-TB regimen, HIV testing date, TB treatment start date, ART start date and County.

Secondary variables were generated from these variables. Age was categorised into 15–19, 20–24, 25–29, 30–34, 35–39, 40–44 and ≥45 years based on the age distribution in the dataset. The BMI variable was categorised using the WHO reference categories of <15, 15–18.4, 18.5–24.9, and ≥25. To determine the timing of ART initiation, the difference in the start date of ART initiation and the start of TB treatment was calculated. This duration was then categorised into <14 days, 15 to 30 days, 31 to 60 days, 61 to 120 days and >120 days. Additional categories for this variable were ART initiated before TB treatment, ART not initiated and missing ART start date. Time to start ART initiation was considered as a proxy for CD4 cell count with the longer the period before ART initiation the higher the likelihood of a high CD4 cell at start of TB treatment. Similarly, to explore if the temporal sequence of HIV testing (knowledge of HIV status) and TB treatment affected outcome, the difference in HIV testing and start of TB treatment (timing of HIV test to TB treatment) was calculated. This duration was then categorised into more than 6 months, 3 to 6 months before TB treatment, 2 to 3 months before TB treatment, 1 month before TB treatment, ±5 days of starting TB treatment, 1 month after starting TB treatment, 2 to 3 months after starting TB treatment, >3 months after starting TB treatment.

### Data management and analysis

De-identified data were extracted from the TIBU database, cleaned, and exported to Stata v13 (Stata Corp, College Station, TC, USA) for analysis. The primary exposure was gender. Patient characteristics and other exposure variables of interest were stratified by gender and summarized with descriptive statistics. The overall proportion and accompanying 95% confidence intervals (CI) adjusted for clustering at the County level for categorical exposures and the means and standard deviation (SD) for continuous variables are reported.

The primary outcome of interest was TB treatment outcome (all-cause mortality during TB treatment). Our data is from a national database, previous surveys and County epidemiological profiles suggest clustering of data within counties with HIV and TB prevalence as well as socioeconomic status varying across the 47 counties [6]. A shared-frailty cox model was used to account for clustering at County level in both the univariate and multi-variate models. Associations between TB treatment outcome and gender were explored using a multivariable cox proportional hazards model to calculate an adjusted association of gender with all-cause mortality during TB treatment. A stepwise forward selection method was used with the Hosmer-Lemeshow criteria of exposure variables with a P value of <0.2 being used to identify exposure variables to include in the multivariable analysis starting with variables with the strongest association. A likelihood ratio test at P<0.05 significance level was used to determine the exposure variables to keep in the model.

To test whether excluding patients who did not have data on HIV testing date, ART treatment start date and TB treatment start date, a sensitivity analysis including this population was conducted (n = 20549) as the outcome data were completed for these cases. To explore whether excluding patients who did not meet our inclusion criteria, we considered patients who had a definitive treatment outcome (bacteriologically confirmed), had completed treatment or cured to have had a ‘favourable outcome’ while those who failed treatment or were lost to follow-up or died as having an ‘unfavourable outcome’, we conducted a sensitivity analysis including this population (n = 22945). A univariable analysis for all missing data but with complete primary outcome data was also undertaken. Tuberculosis and HIV independently lead to loss of weight. We tested for an interaction between time to starting ART and BMI as a binary variable (low <18.5; normal/high ≥18.5).

To assess if survival varied by gender, Kaplan-Meier survival curves were plotted, and log-rank test was used to look for differences in survival of men and women. To explore for the effect of age, we have stratified the curves by age group.

For the univariable and multivariable cox models, crude, and adjusted hazard ratios (HR) with accompanying 95% CIs and corresponding *P* values are reported.

### Ethics approval and considerations

Ethics approval was obtained from Moi University/Moi Teaching and Referral Hospital Institutional Review and Ethics Committee (IREC)- IREC/2016/35. All individual patient data were fully de-identified, and no identifiers were collected. This was a review of routinely collected program dataset, hence no patient interviews were carried out. Informed consent was not a requirement for this study.

## Results

**Fig 1:**
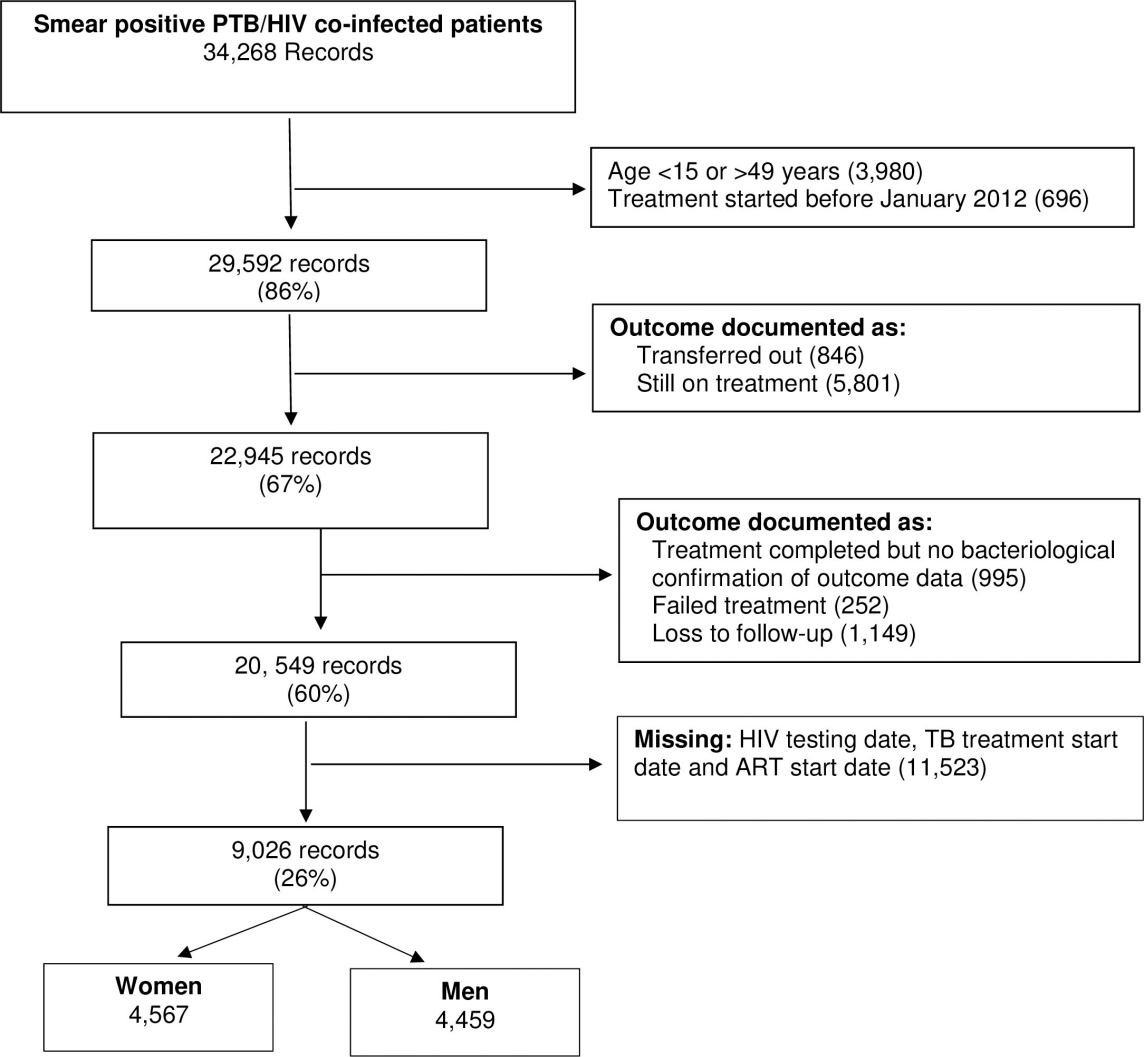
Flow chart showing smear-positive Pulmonary Tuberculosis HIV co-infected patients who were treated for tuberculosis and were eligible for analysis in Kenya, 2012 to 2015.

Records of 34,286 smear-positive PTB HIV co-infected patients were available for analysis for the period 2012–2015. Of these, 9,026 observations met the eligibility criteria and were analysed, out of which 4,567 (50.6%) and 4,459 (49.4%) were women and men, respectively. However, patients who had missing data on HIV testing date, TB treatment start date and ART treatment start date (20,549 observations) were used for sensitivity analysis (**Fig 1**).

**Table 1** shows patient characteristics of patients considered for analysis stratified by gender. Overall, out of the 9,026 patients, 8,154 (90.3%) had their treatment outcome as cured, the mean age in years (SD) was 33.3 (7.5) and the mean body mass index (SD) was 18.2 (3.4). Stratifying by gender, there was a significant association (P<0.05) between gender and all patient characteristics except having a chest radiograph, sputum smear result month 2 and anti-TB regimen. Men had a higher proportion of: deaths 10.6% (471), with a majority being in the older age groups (30% men vs 17% women in those ≥40 years), of those with BMI <18.5 (55.3% men vs 50.6% women) and those who did not have a sputum result at month six 11% (497). Time period between HIV test to TB treatment was longer in women, among those tested for HIV more than 3 months before starting their TB treatment, 29% (1,317) were women compared to 20% (912) in men.

**Table 1:** Descriptive characteristics of smear-positive Pulmonary Tuberculosis HIV co-infected patients who were treated for tuberculosis and were eligible for analysis in Kenya, 2012 to 2015.

Variables	Overall N = 9,026	Women N = 4, 567	Men N = 4, 459	P value
**Treatment Outcome**				
Cured	8,154 (90.3)	4,166 (91.2)	3,988 (89.4)	**0.004**
Died	872 (9.7)	401 (8.8)	471 (10.6)	
**Mean Age in years (SD)**	33.3 (7.5)	31.7 (7.3)	34.9 (7.3)	**<0.001**
**Age group**				
15–24 years	1,059 (11.7)	732 (16.0)	327 (7.3)	
25–29 years	1,865 (20.7)	1,155 (25.3)	710 (15.9)	**<0.001**
30–34 years	2,147 (23.8)	1,089 (23.8)	1,058 (23.7)	
35–39 years	1,827 (20.2)	805 (17.6)	1,022 (22.9)	
40–44 years	1,355 (15.0)	529 (11.6)	826 (18.5)	
45–49 years	773 (8.6)	257 (5.6)	516 (11.6)	
**Mean Body Mass Index [BMI] (SD)**	18.2 (3.4)	18.4 (3.7)	17.9 (2.9)	**<0.001**
**BMI Categories**				
<15	1,093 (12.1)	638 (14.0)	455 (10.2)	**<0.001**
15–18.5	3,681 (40.8)	1,671 (36.6)	2,010 (45.1)	
18.5–24.9	3,081(34.1)	1,622 (35.5)	1,459 (32.7)	
>25	278 (3.1)	207 (4.5)	71 (1.6)	
Missing	893 (9.9)	429 (9.4)	464 (10.4)	
**Chest Radiograph**				
No	8,235 (91.2)	4,142 (90.7)	4,093 (91.8)	0.065
Yes	791 (8.8)	425 (9.3)	366 (8.2)	
**Smear sputum result at month 2**				
Negative	7,634 (84.6)	3,888 (85.1)	3,746 (84.0)	0.335
Positive	589 (6.5)	288 (6.3)	301 (6.8)	
No results	803 (8.9)	391 (8.6)	412 (9.2)	
**Smear sputum result at month 6**				
Negative	8,096 (89.7)	4,134 (90.5)	3,962 (88.9)	**0.009**
No results	930 (10.3)	433 (9.5)	497 (11.1)	
**Anti-TB Regimen**				
2RHZ/4RH	13 (0.1)	6 (0.1)	7 (0.2)	0.936
2RHZE/4RH	8,802 (97.5)	4,455 (97.5)	4,347 (97.5)	
2SRHZE/1RHZE/5RHE	1,10 (1.2)	54 (1.2)	56 (1.3)	
Other	10 (0.1)	4 (0.1)	6 (0.1)	
Missing	91 (1.0)	48 (1.1)	43 (1.0)	
**Time of HIV testing to start of TB treatment**				
More than 6 months before TB treatment	1,863 (20.6)	1,108 (24.3)	755 (16.9)	**<0.001**
3 to 6 months before TB treatment	366 (4.1)	209 (4.6)	157 (3.5)	
2 to 3 months before TB treatment	664 (7.4)	350 (7.7)	314 (7.0)	
1 month before TB treatment	1,236 (13.7)	557 (12.2)	679 (15.2)	
5 days before or after HIV test	4,011(44.4)	1,913 (41.9)	2,098 (47.1)	
1 Month after TB treatment	586 (6.5)	294 (6.4)	292 (6.5)	
2–3 months after TB treatment	215 (2.4)	94 (2.1)	121 (2.7)	
More than 3 months after TB treatment	85 (0.9)	42 (0.9)	43 (1.0)	
**Time of ART start after TB treatment**				
<14 days	2,084 (23.1)	1,010 (22.1)	1,074 (24.1)	**<0.001**
15 to 30 days	1,198 (13.3)	579 (12.7)	619 (13.9)	
31 to 60 days	910 (10.1)	441 (9.7)	469 (10.5)	
More than 60 days	667 (7.4)	357 (7.8)	310 (7.0)	
Before TB treatment	2,620 (29.0)	1,455 (31.9)	1,165 (26.1)	
ART not started	1,547 (17.1)	725 (15.9)	822 (18.4)	

SD = Standard Deviation; S = Streptomycin; R = Rifampicin; H = Isoniazid; Z = Pyrazinamide; E = Ethambutol; TB = Tuberculosis; ART = Antiretroviral Therapy

**Table 2** shows the univariable and multivariable analysis for the association between treatment outcome and gender. The variables chest radiograph, regimen, and sputum result at month 6 had very few subjects when cross-tabulated with the outcome across the different clusters and were excluded from the univariable and multivariable analysis due to challenges in convergence or reliable estimates. In the univariable analysis, there was significant crude association between gender and all-cause mortality during TB treatment. Women had a significantly reduced risk of death compared to men of 0.8 times (HR 0.81; 95% CI 0.71–0.93; p = 0.002). The test for interaction between BMI and time to ART initiation was not significant and therefore an interaction term was not included in the multivariable analysis. In the multivariable analysis, age group, BMI, sputum smear results at 2 months, timing of HIV test and time to ART treatment were identified as significant risk factors for mortality. After adjusting for these factors, there was a 17% significant reduction in the risk of death among women (adjusted HR 0.83; 95% CI 0.72–0.96; p = 0.013), however, this was a small difference when compared to the unadjusted HR = 0.81; 95% CI: 0.71–0.93. Compared to those who had a BMI of <15, a higher BMI was a significant predictor of survival while those with a positive smear positive result at month two had a 50% increased risk of death (adjusted HR 1.51; 95% CI 1.00–2.27) compared to those who had a negative result. Similarly, initiation of ART treatment more than 60 days after starting TB treatment when compared to starting at <14 days was a significant predictor of survival (adjusted HR 0.50; 95% CI 0.32–0.77).

**Table 2:** Univariable and multivariable association between mortality and gender, with men as the reference in smear-positive Pulmonary Tuberculosis HIV co-infected patients who were treated for tuberculosis and were eligible for analysis in Kenya, 2012 to 2015 (N = 9026).

Variable	Univariate		Multivariate	
	HR (95% CI)	P value	aHR (95% CI)	P value
**Sex**				
Women	**0.81(0.71–0.93)**	**0.002**-	**0.83(0.72–0.96)**	**0.013**-
Men	Ref		Ref	
**Age group**				
15–24 years	Ref	0.002	Ref	<0.001
25–29 years	1.05 (0.80–1.39)		1.25 (0.94–1.67)	
30–34 years	**1.36 (1.04–1.76)**		**1.46 (1.11–1.92)**	
35–39 years	**1.4 (1.07–1.82)**		**1.53 (1.16–2.02)**	
40–44 years	**1.53 (1.16–2.02)**		**1.57 (1.17–2.10)**	
45–49 years	**1.51 (1.11–2.06)**		**1.51 (1.08–2.10)**	
**Body Mass Index (BMI) categories**				
<15	Ref	**<0.001**	Ref	<0.001
15–18.5	**0.55 (0.46-.65)**		**0.76 (0.63-.92)**	
18.5–24.9	**0.31 (0.26-.39)**		**0.48 (0.38-.59)**	
>25	**0.22 (0.12–0.40)**		**0.41 (0.22-.77)**	
Missing	**0.68 (0.53-.87)**		**0.78 (0.60–1.01)**	
**Sputum smear result month 2**				
Negative	Ref	**<0.001**	Ref	<0.001
Positive	**1.55 (1.03–2.34)**		**1.51 (1.00–2.27)**	
No results	**61.82 (52.53–72.75)**		**57.72 (48.84–68.22)**	
**Time of HIV test to start of TB treatment**				
More than 6 months before TB treatment	Ref	<0.001	Ref	0.003
3 to 6 months before TB treatment	1.10 (0.76–1.60)		0.99 (.67–1.46)	
2 to 3 months before TB treatment	**1.38 (1.05–1.83)**		1.08 (.81–1.45)	
1 month before TB treatment	**1.63 (1.30–2.04)**		**1.46 (1.13–1.89)**	
5 days before or after HIV test	1.18 (0.97–1.43)		1.04 (.81–1.33)	
1 Month after TB treatment	0.82 (0.57–1.17)		1.03 (.69–1.54)	
2–3 months after TB treatment	0.7 (0.39–1.26)		0.94 (0.50–1.75)	
More than 3 months after TB treatment	0.39 (0.12–1.22)		0.31(0.10–1.01)	
**Time of ART start after TB treatment**				
<14 days	Ref	<0.001	Ref	0.003
15 to 30 days	**0.77 (0.60–0.98)**		0.96 (0.75–1.24)	
31 to 60 days	**0.63 (0.47–0.85)**		0.96 (0.71–1.31)	
More than 60 days	**0.36 (0.23–0.54)**		**0.5 (0.32–0.77)**	
Before TB treatment	0.91 (0.75–1.10)		1.04 (0.83–1.31)	
ART not started	**1.66 (1.36–2.02)**		**1.34 (1.1–1.65)**	

HR = Hazard Ratio; aHR = adjusted Hazard Ratio; CI = Confidence Interval; S = Streptomycin; R = Rifampicin; H = Isoniazid; Z = Pyrazinamide; E = Ethambutol; TB = Tuberculosis; ART = Antiretroviral Therapy

To explore the effect of missing data on our estimates, we included all cases with complete outcome data but missing data on dates in our multivariable analysis (n = 20549; see **Fig 1**). Similar estimates to those in the multivariable analysis in the magnitude and direction of effect were observed in the multivariable sensitivity analysis across all variables (**Table 3**).

**Table 3:** Multivariable sensitivity analysis of association between mortality and gender, with men as the reference in smear-positive Pulmonary Tuberculosis HIV co-infected patients who were treated for tuberculosis and were eligible for analysis in Kenya, 2012 to 2015 (N = 20,549).

Variable	Multivariable analysis	
	aHR (95% CI)	P value
**Sex**		
Women	**0.85 (0.77–0.93)**	**0.001**
Men	Ref	
**Age group**		
15–24 years	Ref	<0.001
25–29 years	1.19 (0.99–1.43)	
30–34 years	**1.30 (1.09–1.56)**	
35–39 years	**1.36 (1.13–1.63)**	
40–44 years	**1.46 (1.21–1.77)**	
45–49 years	**1.39 (1.12–1.72)**	
**Body Mass Index (BMI) categories**		
<15	Ref	<0.001
15–18.5	**0.66 (0.58–0.75)**	
18.5–24.9	**0.47 (0.41–0.55)**	
>25	**0.45 (0.32–0.63)**	
Missing	**0.70 (0.59–0.82)**	
**Sputum smear month 2**		
Negative	Ref	<0.001
Positive	**1.53 (1.15–2.05)**	
No results	**55.96 (50.04–62.58)**	
**Time of HIV test to start of TB treatment**		
More than 6 months before TB treatment	Ref	0.004
3 to 6 months before TB treatment	1.02 (0.72–1.44)	
2 to 3 months before TB treatment	1.03 (0.79–1.33)	
1 month before TB treatment	1.23 (0.98–1.55)	
5 days before or after HIV test	0.95 (0.77–1.17)	
1 Month after TB treatment	0.89 (0.63–1.27)	
2–3 months after TB treatment	0.71 (0.41–1.22)	
More than 3 months after TB treatment	**0.36 (0.15–0.90)**	
Missing ART start date	0.85 (0.69–1.05)	
**Time of ART start after TB treatment**		
<14 days	Ref	<0.001
15 to 30 days	1.00 (0.79–1.26)	
31 to 60 days	0.98 (0.74–1.29)	
More than 60 days	**0.50 (0.34–0.74)**	
Before TB treatment	0.97 (0.79–1.19)	
ART not started	1.30 (1.08–1.55)	

HR = Hazard Ratio; aHR = adjusted Hazard Ratio; CI = Confidence Interval; TB = Tuberculosis; ART = Antiretroviral Therapy

Similarly, estimates from the sensitivity analysis when the outcome was defined as favourable or unfavourable (n = 22 945; see **Fig 1**) were similar in magnitude and direction of effect to those from the complete case data (**S2 Table**). A univariable analysis for the missing data (n = 11 523) is presented in **S1 Table**, no appreciable differences were observed in these data and those included in the main analysis in **Table 2**.

**Fig 2:**
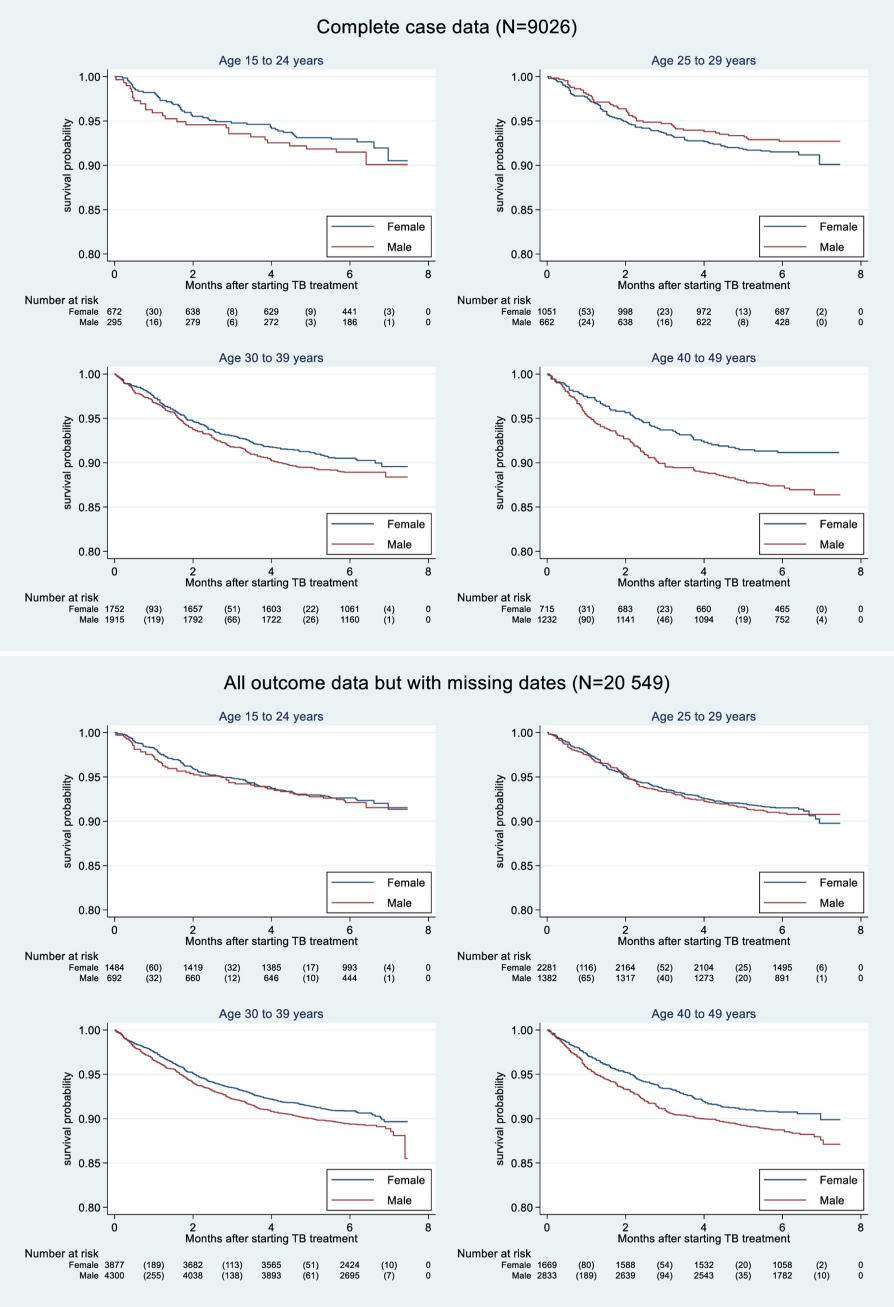
a. Kaplan-Meier plot of survival by gender of smear-positive pulmonary tuberculosis/HIV co-infected patients who were treated for tuberculosis in Kenya, 2012–2015 with complete case record (N-9,026). b. Kaplan-Meier plot of survival by gender of smear-positive pulmonary tuberculosis/HIV co-infected patients who were treated for tuberculosis in Kenya, 2012–2015 with complete outcome data but includes records with missing dates (N = 20, 549).

The survival analysis showed that women had better survival than men, however, there was a significant difference in the 40 to 49 years age group ((log-rank p = 0.006) when using the complete case analysis (**Fig 2A;** N = 9026). Including subjects with complete outcome data, a similar trend was observed (**Fig 2B;** N = 20549) but a significant difference was observed in the age groups 30 to 39 years (log-rank p = 0.037) and 40 to 49 years (log-rank p = 0.018).

To validate the non-significant gender mortality difference in the age group 15 to 24 years subset of patients, we applied, in a sub-analysis **(S3 Table)**, the univariable and multivariable models to this sub-group. While women had decreased risk of death there was no significant difference in this risk aHR 0.78(0.56–1.11).

## Discussion

This study showed that smear positive PTB/HIV co-infected women of reproductive age treated for TB between 2012 and 2015 in Kenya, had a 17% significantly reduced risk of all-cause mortality during TB treatment compared to their men counterparts in the multivariable analysis. Adjusting for potential confounders led to only a small change in the observed reduction suggesting that, alternative explanations should be explored. The finding is consistent with previous studies that reported that women had a higher likelihood of better TB treatment outcomes compared to men [17–20]. A previous study from Kenya carried out in a similar setting, reported worse PTB treatment outcome among women compared to men [6] although the study population was not limited to sputum positive PTB/HIV co-infected patients as done in the current study. However, age-stratified Kaplan-Meier survival curves were significant in the older age group of 40 to 49 years. When all outcome data were used, the Kaplan-Meier survival curves were still significant in the older age groups of 30 to 39 and 40 to 49 years. Similar age-specific gender difference was reported in a HIV patient cohort from Kenya [21].

The significant difference in the older age groups suggest the possible role of poor health seeking behaviour and adherence to treatment among men reported from HIV studies [21]. Applying the univariable and multivariable models in a sub-analysis for the age group 15 to 24 years, indicate no gender difference, supporting the Kaplan-Meier findings and our proposition of behavioural contribution to the observed difference in the older age groups. Our data indicate that more men compared to women were older and had a lower BMI than the women. Older age at presentation, more co-morbidity, limited access to health services and poor adherence are associated with mortality among older men [17, 18, 22–25]. Findings from this study are consistent with previous research which showed that patients with a higher BMI had a lower risk of death [23, 26–28]. Though not explored in this study, differences in biological factors between men and women could explain the higher mortality in men during TB treatment. Female sex hormone oestradiol has been shown to enhance macrophage activation while the male hormone androgen does not [29, 30].

More women compared to men had their HIV test early and were started on ART before TB treatment. This implies that, women were more likely tested for HIV when their CD4 cell counts were higher than 500 cells/μl, which was the cut off for starting ART at the time of study. This can be attributed to women of reproductive age having more interactions with the health system when seeking services for family planning, antenatal care, delivery, postnatal care, and child health clinics [31].

Overall, those initiated on ART at more than 60 days after anti-TB treatment initiation had significantly better survival. Patients who had not started ART had the worst survival. While the current data suggest that treatment initiation should be delayed, this may not be the case. The time to ART initiation variable was used as a proxy to CD4 cell count. Patients who took a longer duration to ART initiation, were also likely to have higher CD4 cell count at initiation of TB treatment. On this presumption, findings from this study are consistent with the wider literature, on early initiation of ART during TB treatment, which leads to a reduction in mortality [7, 9]. However, CD4 cell count and viral load data are not routinely collected in the TIBU database, therefore, difficult to test this assumption.

Sputum smear conversion to negative after two months of TB treatment is the goal of TB programs. Patients who fail to convert after two months risk treatment failure and continue to be infectious [32]. They are also at risk of developing multidrug-resistant TB [33], or they may have had multidrug-resistant TB from the outset. However, multidrug-resistant TB are captured in a separate database and hence were not included in this analysis. Our findings highlight that a positive sputum smear at two months of TB treatment is associated with an increased risk of death among smear positive PTB/HIV co-infected patients. The reasons for non-conversion which include treatment interruptions and poor adherence in HIV/PTB co-infected patients should be studied.

The following limitations should be taken into account: data on the patient’s socioeconomic status, multidrug-resistant TB, adherence to treatment or other behavioural components, viral load and CD4 counts were not collected due the retrospective and routine nature of the study. However, time to ART initiation which has been shown to be a good marker (based on the national guidelines during the period of study), was used as a proxy for CD4 count but we cannot preclude misclassification of patients that might have resulted in delays occasioned from health facilities where HIV and TB services are not integrated. Further, our sub-analysis of the age 15 to 24 years may be under-powered to detect a gender difference, but the direction of effect is supportive of females having a better survival. The strengths of this study include analysis by gender, limiting analysis to only reproductive age and sputum positive PTB/HIV co-infected patients but also undertaking a sensitivity analysis. While we demonstrate the value of routine data in supporting TB/HIV programming to address potential gender disparities we also highlight gaps in data quality and completeness which needs strengthening to better inform decisions and optimise data use. This study also adhered to the STROBE reporting guidelines [34].

The reasons for better survival in women should be explored, and the results used to improve survival in men, while we suggest a behavioural explanation, routine collection of valuable data such as treatment adherence, CD4 counts, viral loads among others should be considered by national TB programmes. Mechanisms of early testing and diagnosis for HIV and TB in men should be explored. Since counties are heterogeneous; social, environmental, and cultural gender disparities, need to be cross-examined.

## Conclusion

Women with sputum positive PTB/HIV co-infection have a significantly lower risk of all-cause mortality during TB treatment compared to men. Men in the study were older, had lower BMI and tested later for HIV. Gender disparities in survival in PTB/HIV co-infected patients should be evaluated further, and TB nutrition programs should be strengthened. To maximise use and value of routine data, more investments to support data quality are required.

## Supporting information

S1 Table Univariable analysis of association between mortality outcome and gender, with men as the reference in smear-positive Pulmonary Tuberculosis HIV co-infected patients who were treated for tuberculosis and were eligible for analysis in Kenya, 2012 to 2015 for patients excluded from the analysis due to missing dates or outcome data not bacteriologically confirmed (N = 11,523). (DOCX)

S2 Table Multivariable sensitivity analysis of association between mortality outcome (favourable vs non-favourable) and gender, with men as the reference in smear-positive Pulmonary Tuberculosis HIV co-infected patients who were treated for tuberculosis and that had outcome data and eligible for analysis in Kenya, 2012 to 2015 (N = 22,945). (DOCX)

S3 Table Multivariable association between mortality outcome and gender, with men as the reference in smear-positive Pulmonary Tuberculosis HIV co-infected patients who were treated for tuberculosis and aged 15 to 24 years in Kenya, 2012 to 2015. (DOCX)
