# Supplementary material for: Gender difference in mortality among pulmonary tuberculosis HIV co-infected adults aged 15-49 years in Kenya
Source: PLoS One. 2020 Dec 14;15(12):e0243977. doi: 10.1371/journal.pone.0243977 (PMC7735576; doi:10.1371/journal.pone.0243977)
Supplement: S1 Table — (DOCX) [file pone.0243977.s001.docx]

| **S1 Table: Univariable analysis of association between mortality outcome and gender, with men as the reference in smear-positive Pulmonary Tuberculosis HIV co-infected patients who were treated for tuberculosis and were eligible for analysis in Kenya, 2012 to 2015 for patients excluded from the analysis due to missing dates or outcome data not bacteriologically confirmed (N=11,523)** | | | |
| --- | --- | --- | --- |
| **Variable** | **Crude HR** | **95% CI** | **P value** |
| **Sex**  Women  Men | 0.85  1.00 | 0.75-0.96  - | 0.011 |
| **Age group**  15 - 24 years  25 - 29 years  30 - 34 years  35 - 39 years  40 - 44 years  45 - 49 years | 1.00  1.28  1.33  1.39  1.41  1.41 | -  1.00-1.63  1.05-1.69  1.09-1.77  1.10-1.81  1.07-1.87 | 0.097 |
| **Body Mass Index (BMI) categories**  <15  15 - 18.5  18.5 - 24.9  >25  Missing | 1.00  0.55  0.37  0.37  0.64 | -  0.46-0.66  0.31-0.45  0.25-0.56  0.52-0.79 | <0.001 |
| **Sputum smear month 2**  Negative  Positive  No results | 1.00  1.61  60.25 | -  1.07-2.41  51.85-70.03 | <0.001 |
| **Time of ART start after TB treatment**  <14 days  15 to 30 days  31 to 60 days after  More than 60 days  Before TB Rx  ART not started  Missing ART start date | 1.00  1.27  1.47  0.41  0.86  0.84  1.43 | -  0.57-2.83  0.64-3.35  0.13-1.25  0.43-1.73  0.48-1.45  0.81-2.54 | <0.001 |
| **Time of HIV test to start of TB treatment**  More than 6 months before TB treatment  3 to 6 months before TB treatment  2 to 3 months before TB treatment  1 month before TB treatment  5 days before or after HIV test  1 Month after TB treatment  2 - 3 months after TB treatment  More than 3 months after TB treatment  Missing ART start date | 1.00  1.63  1.49  0.93  0.96  0.55  0.54  0.60  1.06 | -  0.76-3.50  0.82-2.71  0.51-1.69  0.61-1.51  0.24-1.28  0.16-1.79  0.14-2.54  0.71-1.59 | 0.279 |
| *HR=Hazard Ratio; aHR=adjusted Hazard Ratio; CI= Confidence Interval; TB=Tuberculosis; ART=Antiretroviral Therapy* | | | |
