# Supplementary material for: Gender difference in mortality among pulmonary tuberculosis HIV co-infected adults aged 15-49 years in Kenya
Source: PLoS One. 2020 Dec 14;15(12):e0243977. doi: 10.1371/journal.pone.0243977 (PMC7735576; doi:10.1371/journal.pone.0243977)
Supplement: S2 Table — (DOCX) [file pone.0243977.s002.docx]

| **S2 Table: Multivariable sensitivity analysis of association between mortality outcome (favourable vs non-favourable) and gender, with men as the reference in smear-positive Pulmonary Tuberculosis HIV co-infected patients who were treated for tuberculosis and that had outcome data and eligible for analysis in Kenya, 2012 to 2015 (N=22,945)** | | | |
| --- | --- | --- | --- |
|  | aHR | 95% CI | P value |
| **Gender**  Female  male | 0.86  1.00 | 0.80-0.93  - | <0.001 |
| **Age group**  15 - 24 years  25 - 29 years  30 - 34 years  35 - 39 years  40 - 44 years  45 - 49 years | 1.00  1.07  1.08  1.01  1.10  1.08 | -  0.94-1.22  0.95-1.23  0.88-1.15  0.96-1.27  0.92-1.27 | <0.001 |
| **Body Mass Index (BMI) categories**  <15  15 - 18.5  18.5 - 24.9  >25  Missing | 1.00  0.70  0.57  0.48  0.61 | -  0.63-0.77  0.51-0.63  0.37-0.62  0.54-0.69 | <0.001 |
| **Sputum smear month 2**  Negative  Positive  No results | 1.00  3.84  28.90 | -  3.35-4.40  26.60-31.40 | <0.001 |
| **Time of ART start after TB treatment**  <14 days  15 to 30 days  31 to 60 days after  More than 60 days  Before TB treatment  ART not started  Missing data | 1.00  1.03  0.96  0.64  1.05  1.03  1.35 | -  0.86-1.23  0.78-1.19  0.48-0.84  0.89-1.24  0.90-1.19  1.18-1.56 | <0.001 |
| **Time of HIV test to start of TB treatment**  More than 6 months before TB treatment  3 to 6 months before TB treatment  2 to 3 months before TB treatment  1 month before TB treatment  5 days before or after HIV test  1 Month after TB treatment  2 - 3 months after TB treatment  More than 3 months after TB treatment  Missing ART start date | 1.00  1.19  1.16  1.38  1.09  0.94  0.85  0.36  1.00 | -  0.91-1.57  0.94-1.43  1.15-1.65  0.92-1.28  0.73-1.21  0.59-1.23  0.17-0.77  0.85-1.18 | <0.001 |
| *aHR=adjusted Hazard Ratio; CI= Confidence Interval; TB=Tuberculosis; ART=Antiretroviral Therapy* | | | |
