# Supplementary material for: Gender difference in mortality among pulmonary tuberculosis HIV co-infected adults aged 15-49 years in Kenya
Source: PLoS One. 2020 Dec 14;15(12):e0243977. doi: 10.1371/journal.pone.0243977 (PMC7735576; doi:10.1371/journal.pone.0243977)
Supplement: S3 Table — (DOCX) [file pone.0243977.s003.docx]

| **S3 Table: Multivariable association between mortality outcome and gender, with men as the reference in smear-positive Pulmonary Tuberculosis HIV co-infected patients who were treated for tuberculosis and aged 15 to 24 years in Kenya, 2012 to 2015** | | | |
| --- | --- | --- | --- |
| **Variable** | **aHR** | **95% CI** | **P-value** |
| **Gender**  Female  Male | 0.78  1.00 | 0.56 - 1.11  - | 0.167 |
| **Body Mass Index (BMI) categories**  <15  15 - 18.5  18.5 - 24.9  >25  Missing | 1.00  0.53  0.48  0.59  0.69 | -  0.35 - 0.81  0.30 -0.78  0.08-4.46  0.41-1.18 | <0.001 |
| **Sputum smear month 2**  Negative  Positive  No results | 1.00  1.74  76.67 | -  0.68 -4.43  52.14 -112.75 | 0.046 |
| **Time of ART start after TB treatment**  <14 days  15 to 30 days  31 to 60 days after  More than 60 days  Before TB Rx  ART not started  Missing ART start date | 1.00  0.56  0.96  0.46  0.48  0.55  0.36 | -  0.27-1.18  0.39-2.34  0.15-1.41  0.24-0.95  0.30-0.99  0.19-0.68 | 0.187 |
| **Time of HIV test to start of TB treatment**  More than 6 months before TB treatment  3 to 6 months before TB treatment  2 to 3 months before TB treatment  1 month before TB treatment  5 days before or after HIV test  1 Month after TB treatment  2 - 3 months after TB treatment  More than 3 months after TB treatment  Missing ART start date | 1.00  0.20  0.64  0.57  0.83  0.94  0.32  0.36  0.88 | -  0.05-0.75  0.23-1.78  0.22-1.48  0.42-1.63  0.24-3.65  0.09-1.13  0.04-3.11  0.45-1.73 | 0.024 |
| *aHR=adjusted Hazard Ratio; CI= Confidence Interval; TB=Tuberculosis; ART=Antiretroviral Therapy* | | | |
